# Mechanochemical borylation of aryldiazonium salts; merging light and ball milling

**DOI:** 10.3762/bjoc.13.144

**Published:** 2017-07-26

**Authors:** José G Hernández

**Affiliations:** 1 Institute of Organic Chemistry, RWTH Aachen University, Landoltweg 1, D-52074 Aachen, Germany

**Keywords:** aryldiazonium salts, borylation, eosin Y, mechanochemistry, photocatalysis

## Abstract

Merging of photo- and mechanochemical activation permitted studying the role of eosin Y in the borylation of aryldiazonium salts in a ball mill. Simultaneous neat grinding/irradiation of the reactants and the photocatalyst led to the formation of boronates in a molten state. On the other hand, the catalyst-free liquid-assisted grinding/irradiation reaction also led to product formation, featuring a direct photolysis pathway facilitated by substrate–solvent charge-transfer complex formation.

## Introduction

The use of mechanical force to process materials or to induce chemical transformations is perhaps as old as the history of mankind itself [1]. Similarly, from time immemorial light has also been present on earth, being perhaps photosynthesis and visions the most fundamental connections between light and living organisms [2]. However, combining synergistically photo- and mechanical activations in organic synthesis is still challenging despite the enormous potential of having both activation modes acting simultaneously.

In recent years, mechanochemistry, which encompasses the use of mechanical means by milling, grinding, shearing, cavitation or pulling to induce chemical transformations [3] has become fundamental for discovering new chemical reactivity [4–5] and to develop more sustainable syntheses. Typically, mechanochemical reactions by milling are conducted in non-translucent containers (e.g., agate, ceramics, steels, and tungsten carbide). While this diversity of milling media materials enables controlling, for example, the energy input during the mechanical process, it becomes an obstacle for in situ characterization of mechanochemical reactions, or to facilitate synergistic activation types involving, for example, light and mechanical energy. Recently, however, the in situ study of mechanochemical transformations has been accomplished by combining translucent milling vessels made of poly(methyl methacrylate) (PMMA) with powder X-ray diffraction (PXRD) [6–7], Raman spectroscopy [8], or a combination of both techniques [9]. On the other hand, attempts to combine photo- and mechanical activation to favor chemical processes have been mainly explored in the photodimerizations of olefins by manual grinding of the reactants followed by long UV-light exposure [10], or by vortex grinding [11]. However, until now, studies of photocatalyzed mechanochemical reactions involving, for example, metal complexes [12] or organic photocatalysts (PC) [13] has been underexplored [14], despite photocatalysis could clearly benefit from the excellent mixing under neat or liquid-assisted grinding (LAG) [15] conditions. Additionally, in contrast to solution-based methods, reactions by milling do not suffer from solubility restrictions due to the possibility to bring reactants and catalysts of very different solubility, into close proximity for achieving chemical reactivity. This last aspect is foreseen as highly valuable in transformations using low-soluble PCs (e.g., porphyrins) [16] or during the photochemical synthesis or modification of polymers [17].

The aforementioned context makes one wonder about the potential for conducting chemical reactions under simultaneous photo- and mechanical activation. To test this idea, the photocatalyzed borylation of aryldiazonium salts, first reported in solution by Yan and co-workers was selected as a model reaction [18]. In the original study, irradiation for 18 h of a MeCN solution of aryldiazonium salts, bis(pinacolato)diboron (B_2_pin_2_, **2**) and eosin Y with a 25 W visible light lamp led to the corresponding arylboronates in moderate to good yields [18].

## Results and Discussion

**Figure 1:**
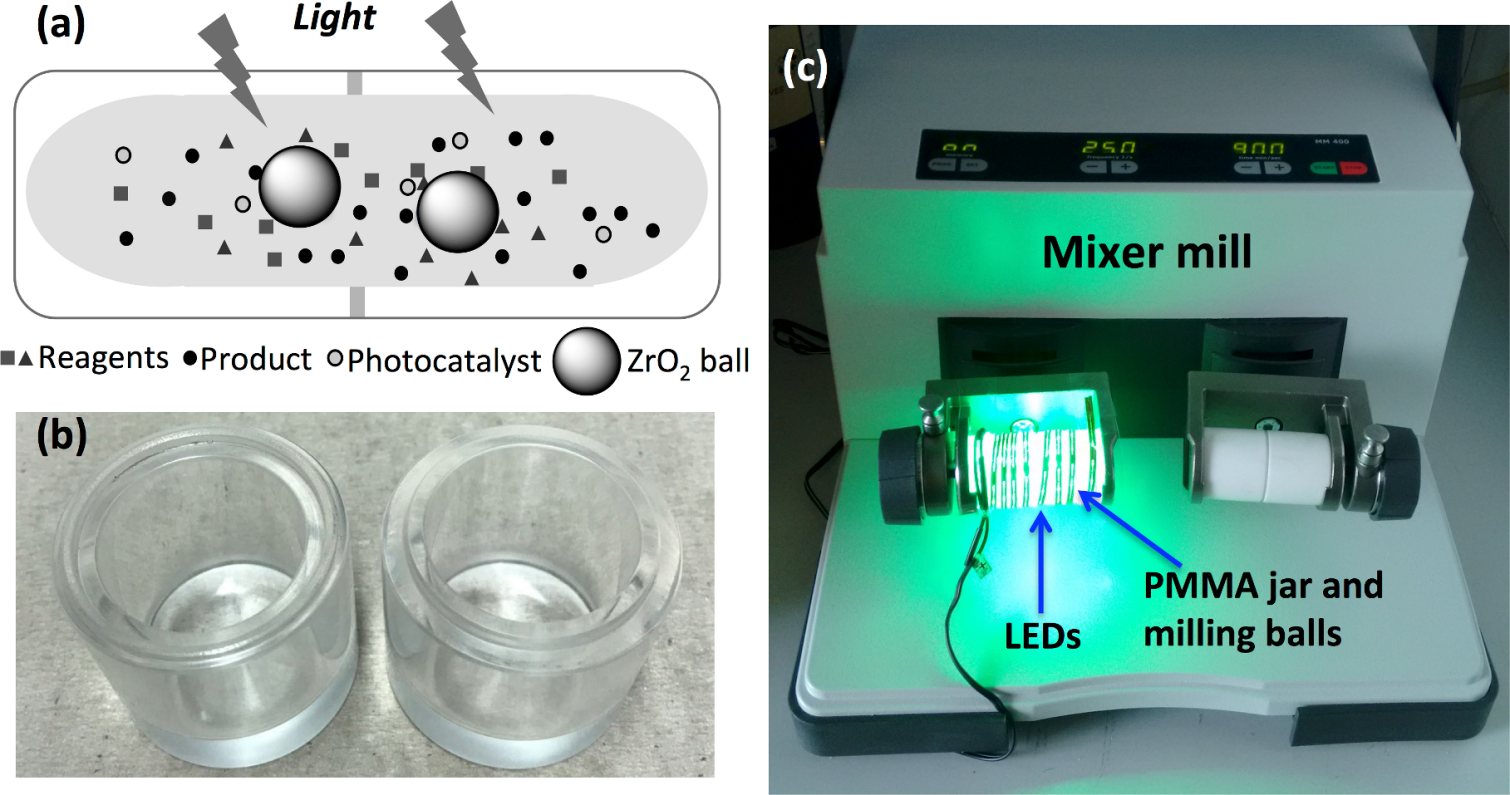
(a) Cartoon representing the merging of light and mechanical energy. (b) 25 mL transparent PMMA milling jar. (c) Experimental setup for simultaneous photo- and mechanical-activation with an external light source.

To commence, a PMMA milling jar was designed to enable external light irradiation of the reaction mixture while having simultaneously the high-speed ball milling acting on the mixture of reactants and PC (Figure 1; for details, see Supporting Information File 1).

Subsequently, with the aim to determine the role of the light, PC and the mechanical milling in the borylation of the aryldiazonium salts, and especially to exclude a potential background borylation reaction triggered by either thermal, mechanical or light-induced heterolytic cleavage of aryldiazonium salts, various control reactions were conducted. First, an equimolar mixture of the diazonium salt **1a** and B_2_pin_2_ (**2**) was milled for 2 h at 25 Hz in a mixer mill, using a Teflon milling jar and ZrO_2_ ball bearings. The safe use of diazonium salt under ball milling conditions has been previously reported in the literature [19]. The analysis of the reaction mixture by ^1^H NMR spectroscopy revealed just the presence of both reactants, both in the presence or absence of the organic photocatalyst eosin Y (5.0 mol %). Ruling out a sole mechanochemical activation pathway (Table 1, entries 1 and 2).

**Table 1:** Screening of the reaction conditions.^a^

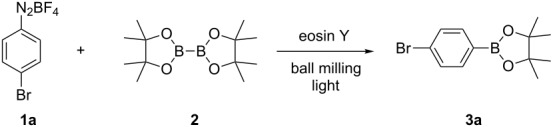				

Entry	Eosin Y (mol %)	Time (h)	Light	**1a**:**3a** (%)^b^

1^c^	–	2	–	100:0
2^c^	(5)	2	–	100:0
3	(5)	2	ambient	100:0
4	–	2	blue LEDs	100:0
5^d^	(5)	2	blue LEDs	94:6
6	(5)	0.5	blue LEDs	83:17
7	(5)	1	blue LEDs	54:46
8	(5)	1.5	blue LEDs	27:73
9	(5)	2	blue LEDs	15:85
10^e^	(5)	2	blue LEDs	59:41
11^f^	(5)	2	blue LEDs	51:49
**12**	**(5)**	**1**	**green LEDs**	**6:94**
13	(0.5)	1.5	green LEDs	63:37

^a^Reaction conditions: a mixture of **1a** (0.369 mmol), **2** (0.369 mmol) and eosin Y was mixed in a 25 mL PMMA milling jar with 15 ZrO_2_ balls of 5 mm in diameter at 25 Hz. ^b^Determined by ^1^H NMR spectroscopy. ^c^A 25 mL Teflon milling jar was used. ^d^**1a**, **2** and the PC were mixed for 30 s in the PMMA jar, then the mixing was stopped and the milling jar was exposed to the light irradiation for 2 h. ^e^The irradiation was stopped after 1 h of reaction. ^f^The milling was stopped after 1 h of reaction.

Repeating the reaction in the presence of the PC in the transparent PPMA milling jar yielded the same negative result proving that ambient light did not mediate the photoredox catalytic borylation reaction under mechanochemical conditions (Table 1, entry 3). Furthermore, neat grinding of a catalyst-free mixture of **1a** and **2** under blue LEDs (light-emitting diodes) light did not afford the borylated product **3a**. In addition to the ^1^H NMR analysis in solution, this result was confirmed by immediate ex situ analysis using IR spectroscopy of the solid reaction mixture, which revealed only the presence of both starting materials. Thereby, excluding a direct thermal [20–21] or photolysis pathway operating under solventless conditions (Table 1, entry 4). Then, a premilled mixture of **1a**, B_2_pin_2_, and eosin Y was subjected to irradiation with blue LEDs for 2 h in the absence of milling (Table 1, entry 5). After the irradiation was halted, the reaction mixture was immediately analyzed by ^1^H NMR spectroscopy. This time trace quantities of product **3a** were detected (Table 1, entry 5). This interesting result under solvent-free conditions encouraged performing the light irradiation accompanied by milling to improve mixing and to increase the surface exposure of the reaction mixture. In a following set of experiments, milling of the reactants and PC was carried out for a time in the range of 15 min to 2 h. The analysis of the composition of the reaction mixture showed significant formation of the product after 30 min of milling/irradiation (Table 1, entry 6). Monitoring the progress of a mixture of the reactants and PC in CD_3_CN at room temperature by ^1^H NMR spectroscopy over a period of time of 20 h showed a composition (98:2; **1a**:**3a**), ruling out the formation of the **3a** during the standard analysis time. Furthermore, the presence of **3a** in the mixture coincided with the observation of an initial molten state of the mixture inside the milling jar [22]. This more homogeneous mixture could have increased the mobility of the reactants favoring the SET process. Reaching the molten state clearly required having both activation modes acting simultaneously, since only milling of **1a** (mp 138–141 °C), **2** (mp 139–140 °C) and eosin Y (mp 305–307 °C), or just irradiation of the mixture did not lead to an observable melting of the solids (Table 1, entries 2–4 and 5). Besides, propagation of this molten state could have been favored by the gradual rise in concentration of the lower-melting product **3a** (mp 69–70 °C) in the mixture. Indeed, milling the product **3a** under the standard milling conditions using the LEDs led to its melt. Similarly, milling a mixture of **1a**, **2** and **3a** for 1 h under light irradiation reached a eutectic melt phase. The need for simultaneous light and mechanical milling was also confirmed after conducting experiments for 2 h where either the milling or the irradiation was stopped after the first hour. In both cases the outcome of the reaction gave similar results compared to having both energy sources acting together for 1 h (Table 1, entries 7, 10, and 11).

**Scheme 1:**
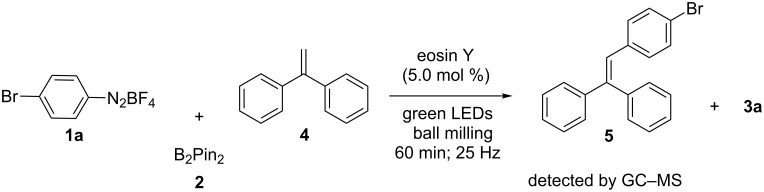
Borylation of **1a** in the presence of 1,1-diphenylethene (**4**).

Next, further tuning of the reaction conditions revealed green LEDs to be a more efficient light source for the reaction with eosin Y (for details, see Table S1 in Supporting Information File 1). This change permitted the transformation to take place after 1 h of milling/irradiation. Under these conditions, the ratio **1a**:**3a** in the reaction mixture reached 6:94 (entry 12 in Table 1). Alternatively, longer reaction times allowed reducing the amount of the organic photocatalyst to 1.0 mol % and 0.5 mol % (entry 13 in Table 1; for more details, see Table S1 in Supporting Information File 1). Then, using the green LEDs an experiment in the presence of 1,1-diphenylethene (**4**) as a radical inhibitor was conducted. After the standard 1 h of milling, the formation of **3a** was slowed down and the analysis of the reaction mixture by gas chromatography–mass spectrometry showed the presence of the phenyl radical trapping adduct **5** (Scheme 1).

With the optimized reaction conditions in hand, we explored the photomechanochemical borylation of the halogenated aryldiazonium salts **1a**–**d** (Table 2).

**Table 2:** Borylation of aryl diazonium salts **1** with **2**.^a^

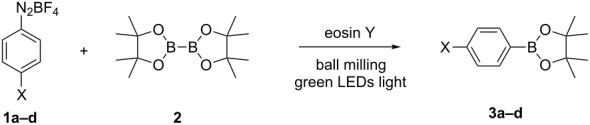				

Entry	Aryldiazonium salt	Product	Time (min)	Yield (%)^b^

1	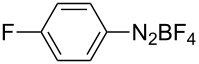 **1b**	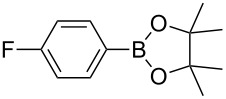 **3b**	90	60
2	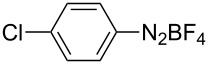 **1c**	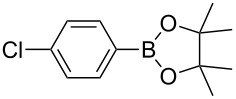 **3c**	45	55
3	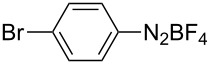 **1a**	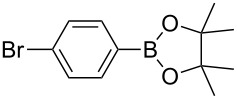 **3a**	60	68
4	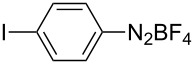 **1d**	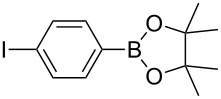 **3d**	120	41
5	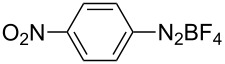 **1e**	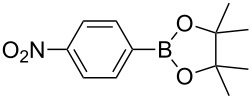 **3e**	120	49

^a^Reaction conditions: a mixture of **1** (0.369 mmol), **2** (140.6 mg; 0.554 mmol) and photocatalyst (5 mol %) was mixed in a 25 mL PMMA milling jar with 15 ZrO_2_ balls of 5 mm in diameter at 25 Hz. ^b^After column chromatography.

Analogously to the case of **1a**, the fluoro and chloro substituted aryldiazonium salts **1b** and **1c** did react affording the boronates **3b** and **3c** (Table 2, entries 1 and 2). It was noticed, however, that the milling/irradiation time required for these substrates to react varied in comparison with the reaction of **1a**. Furthermore, attempts to react the 4-iodobenzenediazonium tetrafluoroborate (**1d**) with **2** were made with low success even after 2 h of reaction time (Table 2, entry 4). As indicated above, the development of a molten state upon irradiation and milling appears to be a key prerequisite for the photomechanochemical borylation to occur [14,22]. Reaching that molten state in the reaction with the iodo derivative **1d** proved challenging, only a part of the reaction mixture seemed homogeneous. Differential scanning calorimetry (DSC) analysis of the aryldiazonium salts **1a**–**d** revealed that **1a**–**c** melt followed by decomposition of the samples. However, the DSC profile of the iodobenzenediazonium salt **1d** showed a direct thermal decomposition upon heating. NMR analysis of the molten **1d** revealed the presence of 1-fluoro-4-iodobenzene (for details, see Supporting Information File 1). Therefore, the difference in melting point temperatures of the substrates and their thermal stability could have a direct correlation with the observed reactivity in the ball mill (Table 2). Control experiments by stirring/heating **1a**–**d** and **2** in an oil bath until the melting of the mixture was reached, showed predominantly thermal decomposition of the aryldiazonium salts **1a**–**d** into the 1-halo-4-fluorobenzene derivatives [23] and only in some cases trace quantities of **3a**–**d** were detected. Meaning that the external light contributes to both, heating the reaction mixture to its eutectic and it also initiated the photoredox process. Similarly, a photocatalyzed reaction between **2** and 4-nitrobenzenediazonium tetrafluoroborate (**1e**), a salt found also to undergo decomposition at 423 K, turned out difficult (for the DSC traces of **1a–e** see Supporting Information File 1).

After 2 h of milling/irradiation the corresponding product **3e** was obtained in moderate yield. In general, after the milling/irradiation experiments no aryldiazonium salt was observed in the reaction mixture, however, the moderate yields for the products **3a**–**e**, even in the presence of 1.5 equiv of **2**, could have been the result of background reactions undergone by **1a**–**e** under the reaction conditions, especially due to the rise in temperature observed upon light irradiation.

**Scheme 2:**
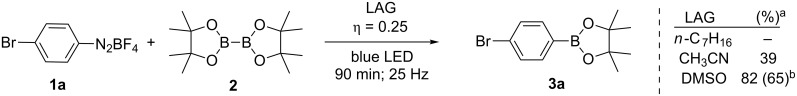
Light-mediated LAG borylation of **1a**. ^a^Determined by ^1^H NMR spectroscopy using internal standard. ^b^After column chromatography.

As mentioned above, LAG, an alternative to the standard neat grinding, has become an useful parameter in mechanochemistry to control chemical selectivity and product composition by having catalytic volumes of organic solvent during the milling [24]. Here, a change in the milling approach from neat to LAG was anticipated to have the potential for switching the activation mode from a SET process to a direct heterolytic photolysis. Jacobi von Wangelin et al. noticed that the borylation product **3a** could also occurred in the absence of eosin Y, upon irradiation of a MeCN solution of the reactants with white LEDs [23,25]. Pleasingly, LAG (MeCN or DMSO η = 0.25) of **1a** and **2** under blue LEDs, and in the absence of eosin Y did also generate the product **3a**. In contrast, LAG experiments using *n*-heptane failed at producing **3a** (Scheme 2; for details, see Table S2 in Supporting Information File 1).

Similarly, control LAG/irradiation experiments conducted in a Teflon milling jar, only formed trace quantities of the product **3a**, ruling out a sole thermal activation of the system by the light source. These results not only illustrates the versatility of mechanochemistry to control the chemical reaction pathway operating in the process, but also sheds light on the role of the photocatalyst in the borylation of the aryldiazonium salts.

Under non-catalyzed LAG/irradiation conditions, charge-transfer complexes between **1a** and appropriate organic solvents could be responsible for the fast generation of the aryl cations in the ball mill, leading to the direct formation of **3a** [26–27]. This observation is in agreement with the findings by Jacobi von Wangelin et al., who described that upon irradiation of a solution of **1a** in MeCN direct heterolytic cleavage of the aryldiazonium salt occurred [23,25]. However, the formation of **3a** under solvent-free milling conditions (vide supra) could have been indeed the result of a photoredox transformation where the organic photocatalyst eosin Y played a key role in triggering the SET process.

## Conclusion

In summary, simultaneous activation of an organic system by light and ball milling techniques has been successfully accomplished for the first time. The utilization of translucent milling vessels permitted the study of the photoborylation of aryldiazonium salts in the presence and in the absence of the organic photocatalyst eosin Y. The results of this proof-of-concept demonstration revealed that under neat grinding conditions the PC does play a role in initiating a SET borylation. Furthermore, the implementation of a LAG/irradiation approach allowed the borylation reaction to occur under catalyst-free conditions. This observation is supported by the tendency of the electrophilic aryldiazonium salts to undergo direct heterolytic photolysis facilitated by organic solvents, upon exposure to near-UV or blue light. In addition to this, the contribution from the increase in temperature experienced during the light exposure and mechanical milling was observed to be vital for the neat grinding, facilitating the formation of molten reaction mixtures.

Despite the still existing technical challenges for merging light and mechanical energy, the positive cooperative synergism between light and mechanical activation reported here, will certainly stimulate the design of more innovative experimental setups [28] and, more important, the exploration of new photomechanochemical organic reactions, where solubility constrains caused by working with low-soluble photocatalysts, substrates or products can be bypassed by mechanochemical means.

## Supporting Information

File 1 Experimental procedures, experimental set-ups and characterization data, NMR spectra, and DSC traces.
