# Supplementary material for: Mechanochemical borylation of aryldiazonium salts; merging light and ball milling
Source: Beilstein J Org Chem. 2017 Jul 26;13:1463–9. doi: 10.3762/bjoc.13.144 (PMC5550817; doi:10.3762/bjoc.13.144)
Supplement: File 1 — Experimental procedures, experimental set-ups and characterization data, NMR spectra, and DSC traces. [file Beilstein_J_Org_Chem-13-1463-s001.pdf]

Supporting Information
for
Mechanochemical borylation of aryldiazonium salts; merging light and ball milling

José G. Hernández*

Address: Institute of Organic Chemistry, RWTH Aachen University, Landoltweg 1, D-52074 Aachen, Germany

Email: José G. Hernández - jose.hernandez@oc.rwth-aachen.de

*Corresponding author

Experimental procedures, experimental set-ups and characterization data, NMR spectra, and DSC traces

CONTENTS

**1. General information**	S2
**2. Milling media and equipment**	S3
**3. General procedures for the mechanochemical reactions**	S4
**4. Table S1. Screening of the reaction conditions; neat grinding**	S5
**5. Table S2. Screening of the reaction conditions; LAG experiments**	S6
**6. Characterization of products 3a–e**	S6
**7. NMR spectra of 3a–e**	S8
**8. Differential Scanning Calorimetry (DSC) of 1a-e**	S16
**9. Thermal decomposition of iodophenyl diazonium tetrafluoroborate (1d)**	S19
**10. Borylation of 1a in the presence of 1,1-diphenylethene (4)**	S19
**11. References**	S21

1. General information

NMR spectra were recorded on a VNMRS 400 or a VNMRS 600 spectrometer. Proton chemical shifts are reported in parts per million on the *δ* scale and are calibrated using the residual non-deuterated solvent signal as an internal reference (MeCN-d_3: δ = 1.94 ppm). Spectral data is provided as follows: chemical shift in ppm (from downfield to upfield), multiplicity (s = singlet, d = doublet, m = multiplet), integration and coupling constant *J*. GC–MS analysis was performed on an Agilent 7890A GC with an Agilent 5975C inert XL EI/CI MSD with triple axis detector and an Agilent DB-5ms column (30 m × 0.25 mm × 0.25 µm) using helium as the carrier gas. detector. Differential scanning calorimetry (DSC) analysis was conducted on a Mettler Toledo DSC System. All samples were heated at a rate of 5 °C/min from 25 to 200 °C. Thermograms were analyzed using Mettler Toledo software.

Mechanochemical reactions were carried out in a RETSCH MM400 Mixer mill using milling jars made of Plexiglas® [(Poly(methyl methacrylate) (PMMA)], or Teflon® with ZrO_2 milling balls of 5 or 1.5 mm in diameter. Blue and green irradiation was provided by LEDs strips of 70 cm and 90 cm in length respectively, coiled around the milling jar. Substrates **1c**, **1b**, **1d** were prepared according to reported methods.[1] Aryldiazonium salts **1a** and **1e** were purchased from commercial suppliers.

2. Milling media and equipment

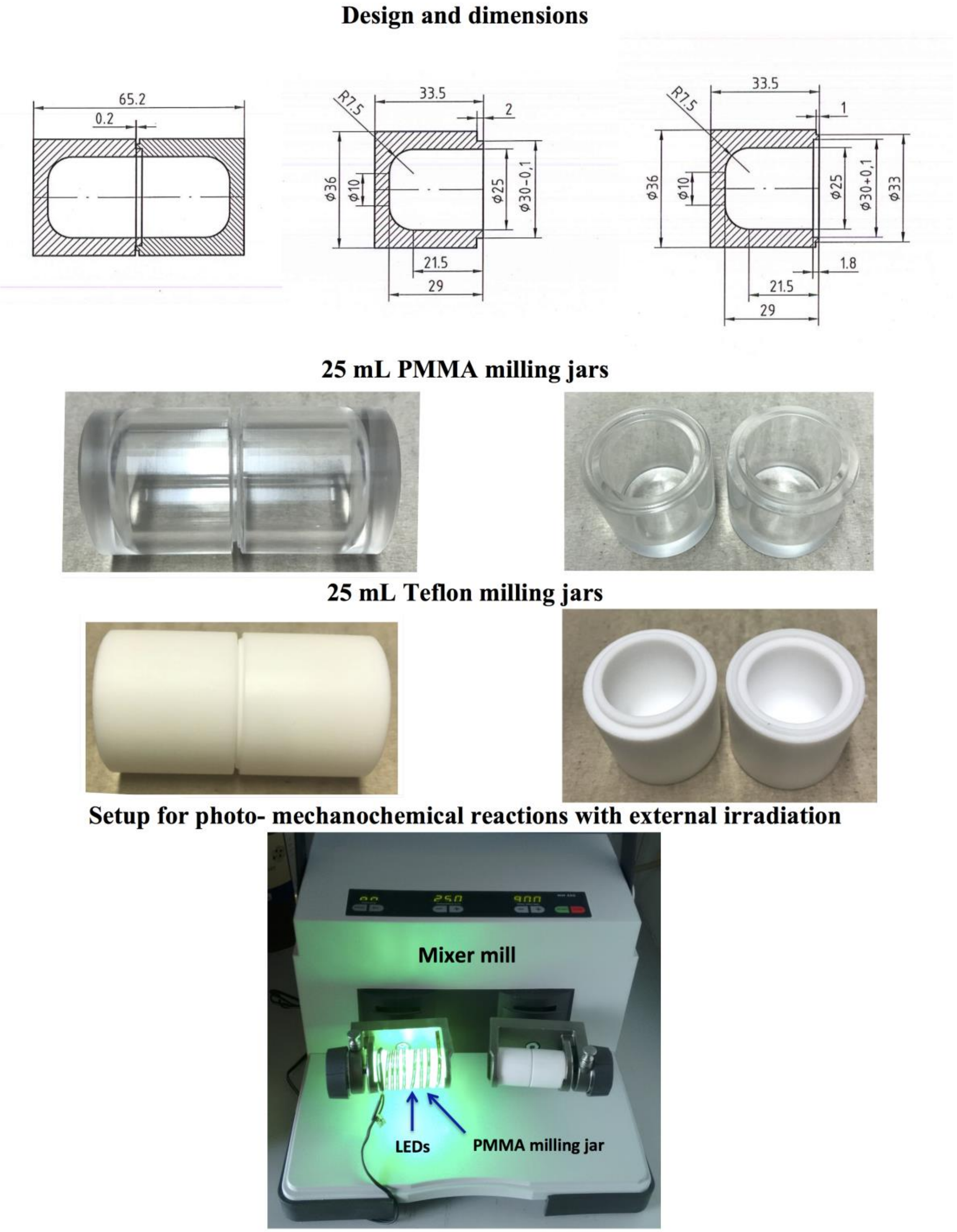

3. General procedures for the mechanochemical reactions

3.1 Neat grinding experiments:

A mixture of **1** (0.369 mmol), **2** (0.369 or 0.554 mmol) and eosin Y (11.96 mg; 5 mol %) was mixed in a 25 mL PMMA milling jar with 15 ZrO_2 balls of 5 mm in diameter at 25 Hz. Irradiation of the reaction mixture was achieved by wrapping the milling jar with a green-LED strip (90 cm; see picture in section 2). After the milling was stopped, the reaction mixture was recovered from the milling jar and the product was purified by column chromatography (SiO_2, eluent 100:1 *n*-pentane/ethyl acetate).

3.2 LAG experiments:

A mixture of **1a** (50 mg; 0.185 mmol) and **2** (70 mg; 0.277 mmol) was mixed in the presence of the solvent (30 μL, $\eta = 0.25$), in a 25 mL PMMA milling jar (higher values of LAG can cause chemical damage to the plastic milling jar). Irradiation of the reaction mixture was achieved by wrapping the PMMA milling jar with a blue-LED strip (70 cm). After the milling was stopped, the reaction mixture was recovered from the milling jar and the product was purified by column chromatography (SiO_2, eluent 100:1 *n*-pentane/ethyl acetate).

4. Table S1. Screening of the reaction conditions; neat grinding.[a]

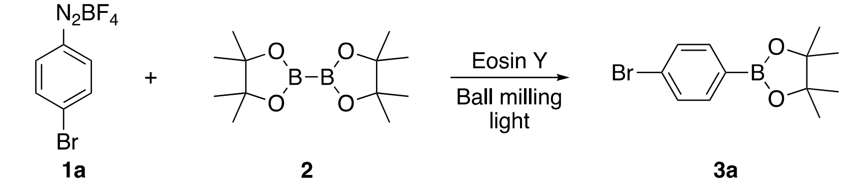

Entry	Eosin Y (mol%)	Time (h)	Light	**1a**:**3a** (%)[f]
1[b]	---	2	---	100:0
2[b]	5	2	---	100:0
3	5	2	ambient	100:0
4	---	2	blue LEDs	100:0
5[c]	5	2	blue LEDs	93:7
6	5	0.5	blue LEDs	83:17
7	5	1	blue LEDs	54:46
8	5	1.5	blue LEDs	27:73
9	5	2	blue LEDs	15:85
10[d]	5	2	blue LEDs	59:41
11[e]	5	2	blue LEDs	51:49
**12**	**5**	**1**	**green LEDs**	**6:94**
13	5	0.5	green LEDs	30:70
14	3	1	green LEDs	20:80
15	1	1	green LEDs	24:76
16	0.5	1.5	green LEDs	63:37

[a] Reaction conditions: a mixture of **1a** (0.369 mmol), **2** (0.369 mmol) and eosin Y was mixed in a 25 mL PMMA milling jar with 15 ZrO_2 balls of 5 mm in diameter at 25 Hz. [b] A 25 mL Teflon milling jar was used. [c] **1a**, **2** and the PC were mixed for 30 s in the PMMA jar, then the mixing was stopped and the milling jar was exposed to the light irradiation for 2h. [d] The irradiation was stopped after 1 h of reaction, [e] The milling was stopped after 1 h of reaction, [f] determined by ^{1}H NMR spectroscopy.

5. Table S2. Screening of the reaction conditions; LAG experiments.[a]

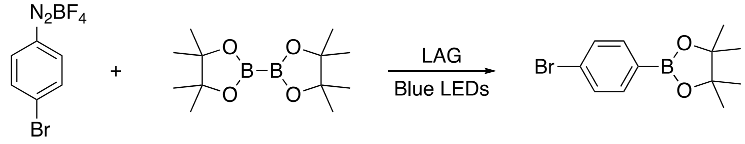

1a 2 3a

Entry	Light	LAG; solvent (η)	Time (h)	1a:3a (%)[b]
**1**	---	---	2	100:0
**2**	Blue LEDs	---	2	100:0
**3**	Blue LEDs	*n*-heptane (0.25 or 0.35)	2	99:1
**4**	Blue LEDs	MeCN (0.35)	2	46:54
**5**	Blue LEDs	DMSO (0.25)	1.5	2:98 (65)[c]
**6**	---	DMSO (0.25)	2	94:6

[a] Reaction conditions: a mixture of **1a** (50 mg; 0.184 mmol), **2** (70 mg; 0.277 mmol) and the LAG solvent was mixed at 25 Hz. [b] Determined by ^{1}H NMR spectroscopy. [c] After column chromatography.

6. Characterization of products 3a–e

2-(4-Fluorophenyl)-4,4,5,5,-tetramethyl-1,3,2-dioxaborolane (3b)[2]

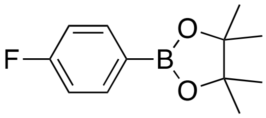

^{1}H NMR (400 MHz, CD_3CN): δ (ppm) 7.81-7.68 (m, 2H), 7.19-7.08 (m, 2H) 1.32 (s, 12H).
^{13}C NMR (100 MHz, CD_3CN): δ (ppm) 165.9 (d, *J* = 248.8 Hz), 137.8 (d, *J* = 8.8 Hz), 115.8 (d, *J* = 20.6 Hz), 85.0, 25.2. The carbon directly attached to the boron atom was not detected, likely due to quadrupolar broadening.
^{11}B NMR (192 MHz, CD_3CN): δ (ppm) 29.3; **^{19}F NMR (376 MHz, CD_3CN)**: δ (ppm) -110.2.
MS (EI) *m/z* = 222.1 [M^+], 207.1, 136.1, 123.0, 85.1.

2-(4-Chlorophenyl)-4,4,5,5,-tetramethyl-1,3,2-dioxaborolane (3c)[3]

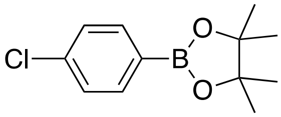

^{1}H NMR (400 MHz, CD_3CN): δ (ppm) 7.69-7.61 (m, 2H), 7.41-7.39 (m, 2H) 1.32 (s, 12H).
^{13}C NMR (100 MHz, CD_3CN): δ (ppm) 137.0, 129.0, 118.3, 85.1, 25.2. The carbon directly attached to the boron atom was not detected, likely due to quadrupolar broadening.
^{11}B NMR (192 MHz, CD_3CN): δ (ppm) 30.6.
MS (EI) *m/z* = 238.1 [M^+], 223.1, 152.0, 139.0, 103.0.

2-(4-Bromophenyl)-4,4,5,5,-tetramethyl-1,3,2-dioxaborolane (3a)[3]

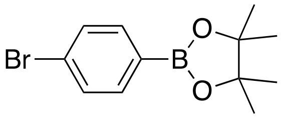

^{1}H NMR (400 MHz, CD_3CN): δ (ppm) 7.62-7.60 (m, 2H), 7.55-7.54 (m, 2H) 1.31 (s, 12H).
^{13}C NMR (100 MHz, CD_3CN): δ (ppm) 137.1, 132.0, 126.6, 85.1, 25.1. The carbon directly attached to the boron atom was not detected, likely due to quadrupolar broadening.
^{11}B NMR (192 MHz, CD_3CN): δ (ppm) 30.6.
MS (EI) *m/z* = 284.0 [M^+], 282.0 [M^+], 267.0, 196.0, 183.0, 103.0.

2-(4-Iodophenyl)-4,4,5,5,-tetramethyl-1,3,2-dioxaborolane (3d)[4]

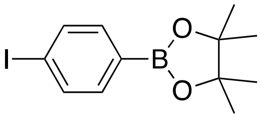

^{1}H NMR (600 MHz, CD_3CN): δ (ppm) 7.80-7.75 (m, 2H), 7.49-7.43 (m, 2H) 1.31 (s, 12H).
^{13}C NMR (150 MHz, CD_3CN): δ (ppm) 138.1, 137.0, 99.1, 85.1, 25.2. The carbon directly attached to the boron atom was not detected, likely due to quadrupolar broadening.
^{11}B NMR (192 MHz, CD_3CN): δ (ppm) 30.7.
MS (EI) *m/z* = 331.1 [M^+], 330.0 [M^+], 329.1 [M^+], 315.0, 244.0, 229.0, 127.0, 104.1.

2-(4-Nitrophenyl)-4,4,5,5,-tetramethyl-1,3,2-dioxaborolane (3e)[3]

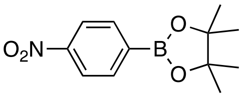

^{1}H NMR (400 MHz, CD_3CN): δ (ppm) 8.22-8.16 (m, 2H), 7.95-7.88 (m, 2H) 1.35 (s, 12H).
^{13}C NMR (100 MHz, CD_3CN): δ (ppm) 150.9, 136.3, 123.5, 85.6, 25.1. The carbon directly attached to the boron atom was not detected, likely due to quadrupolar broadening.
^{11}B NMR (128 MHz, CD_3CN): δ (ppm) 30.1.
MS (EI) *m/z* = 249.1 [M^+], 234.1, 163.1.

7. NMR spectra of 3a–e

^{1}H NMR of 2-(4-Fluorophenyl)-4,4,5,5-tetramethyl-1,3,2-dioxaborolane (3b)

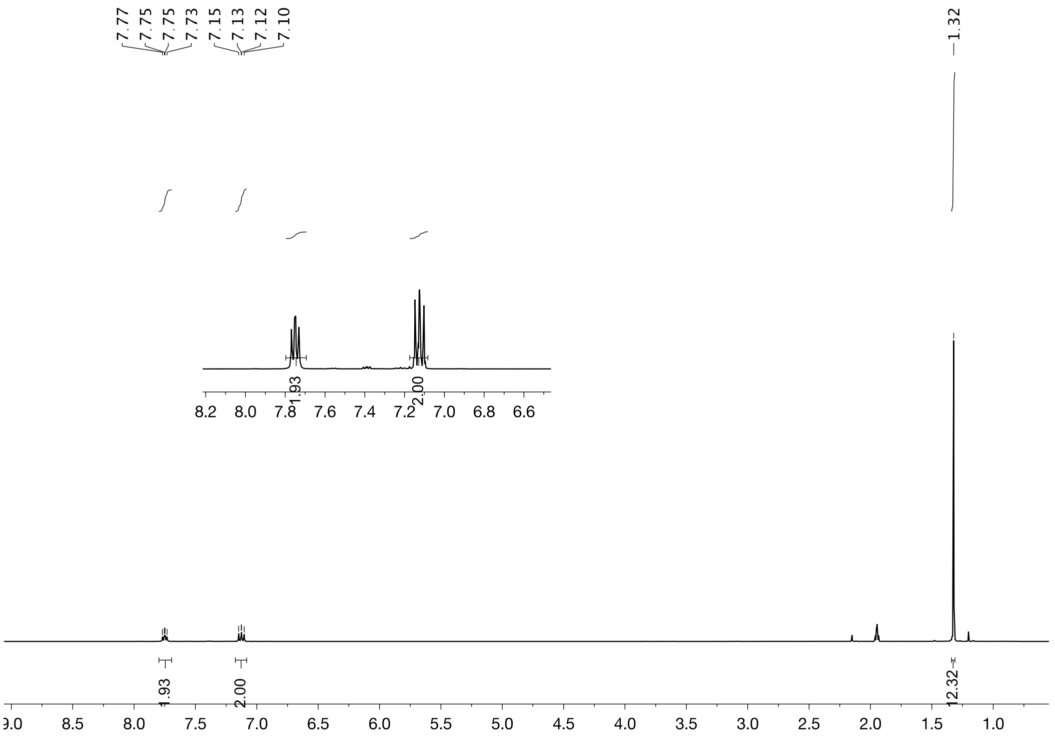

^{13}C NMR of 2-(4-Fluorophenyl)-4,4,5,5-tetramethyl-1,3,2-dioxaborolane (3b)

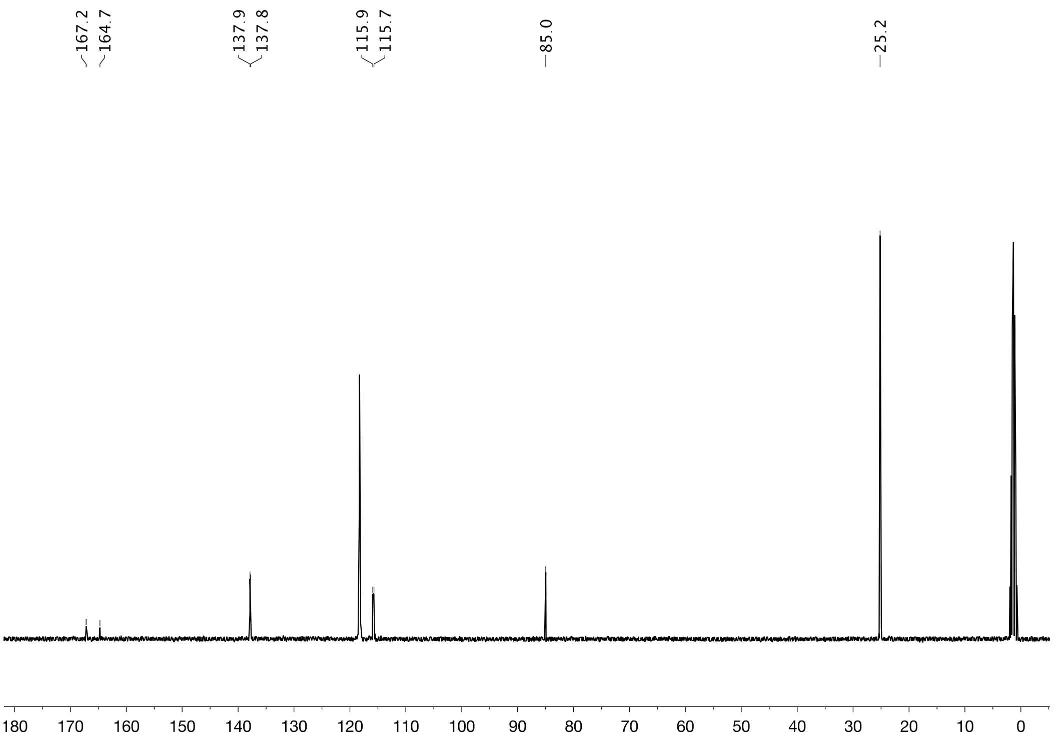

^{11}B NMR of 2-(4-Fluorophenyl)-4,4,5,5-tetramethyl-1,3,2-dioxaborolane (3b)

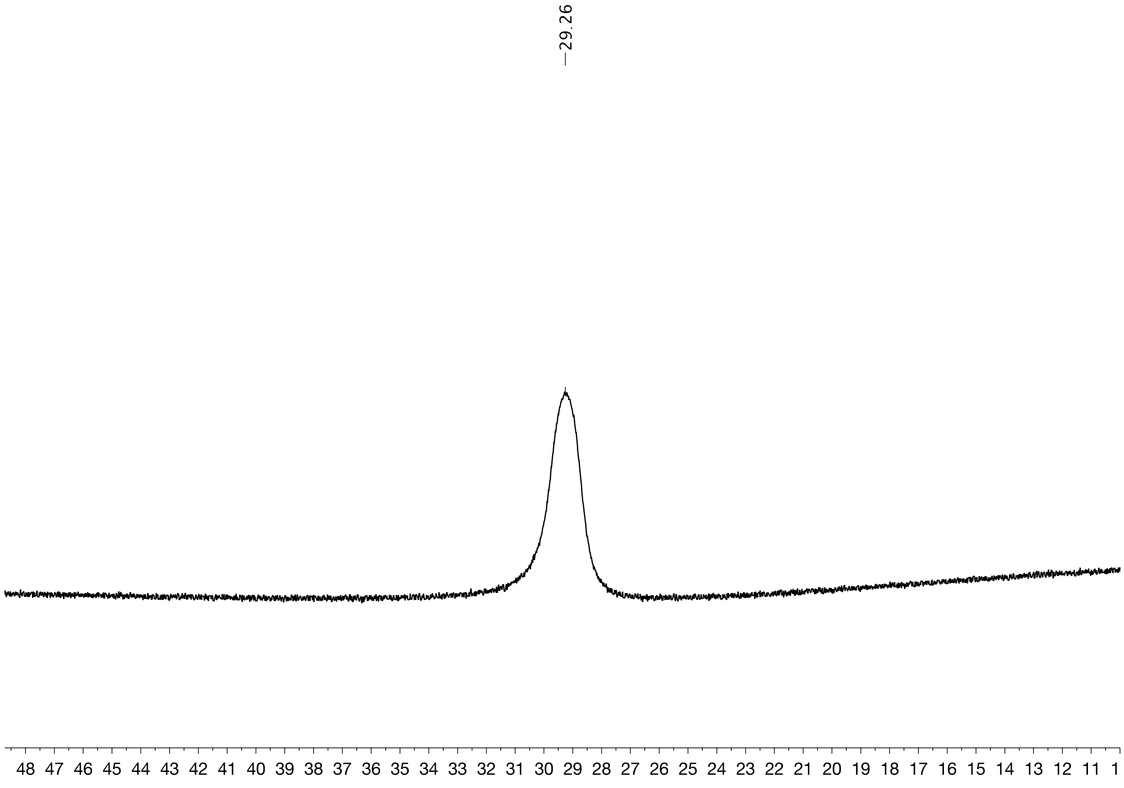

^{19}F NMR of 2-(4-Fluorophenyl)-4,4,5,5-tetramethyl-1,3,2-dioxaborolane (3b)

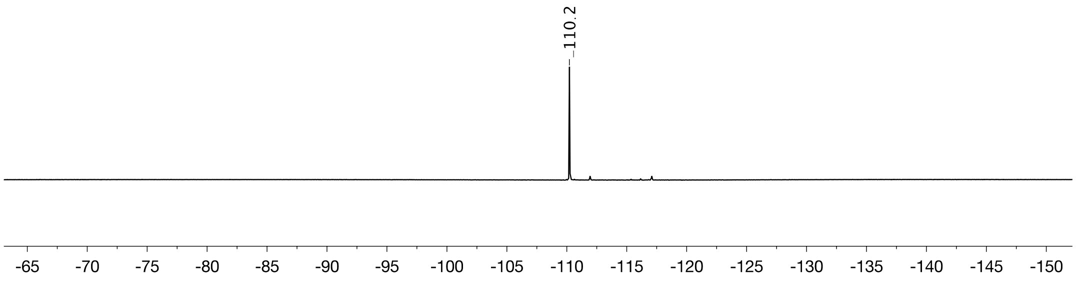

^{1}H NMR of 2-(4-Chlorophenyl)-4,4,5,5-tetramethyl-1,3,2-dioxaborolane (3c)

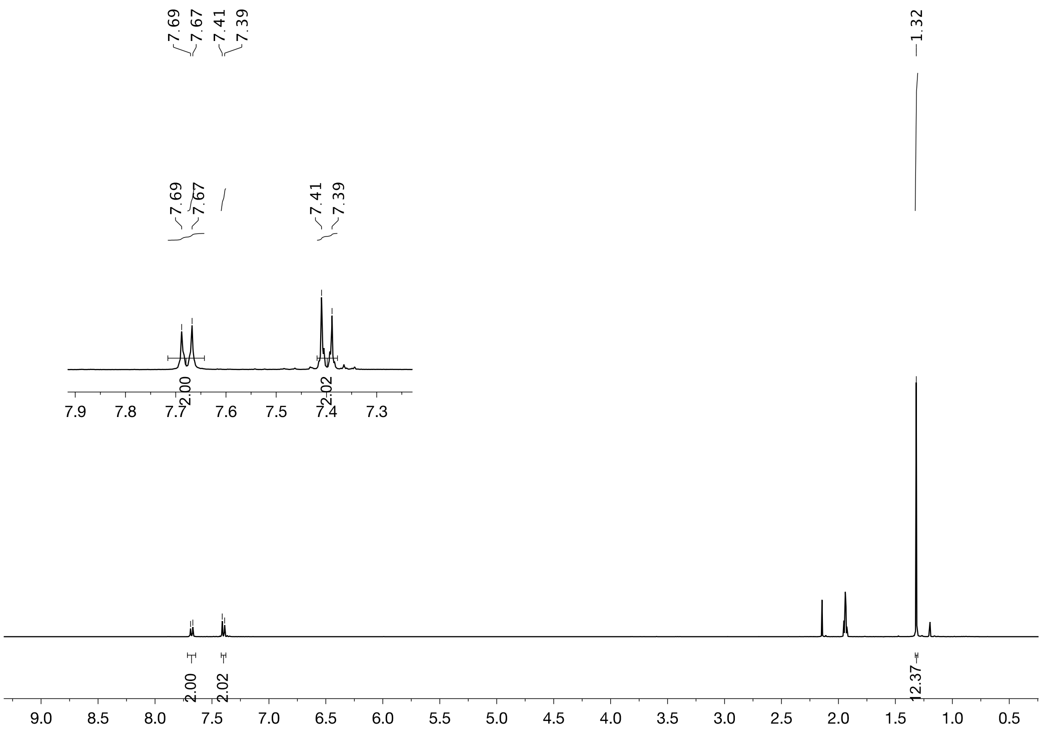

^{13}C NMR of 2-(4-Chlorophenyl)-4,4,5,5-tetramethyl-1,3,2-dioxaborolane (3c)

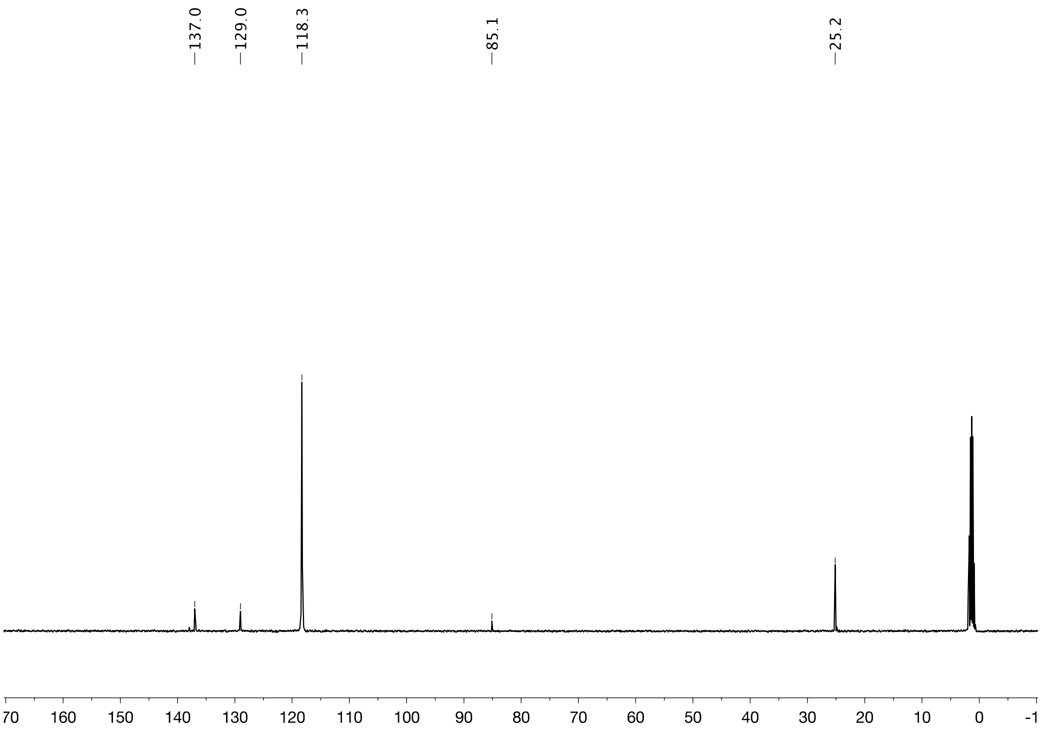

^{11}B NMR of 2-(4-Chlorophenyl)-4,4,5,5-tetramethyl-1,3,2-dioxaborolane (3c)

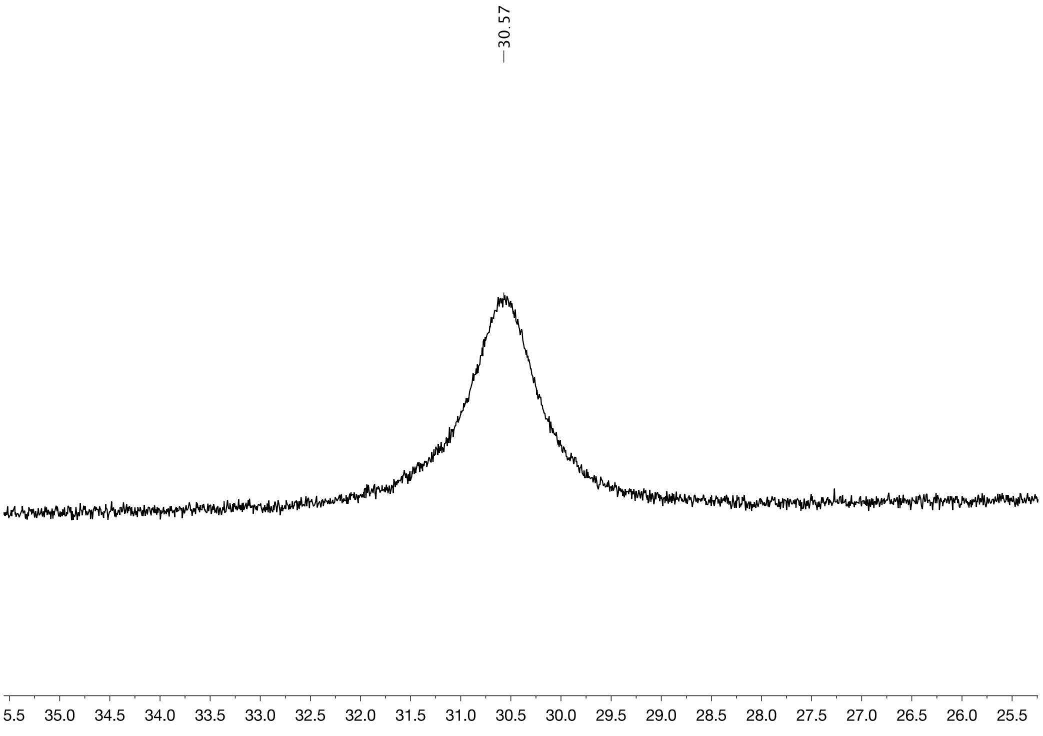

^{1}H NMR of 2-(4-Bromophenyl)-4,4,5,5-tetramethyl-1,3,2-dioxaborolane (3a)

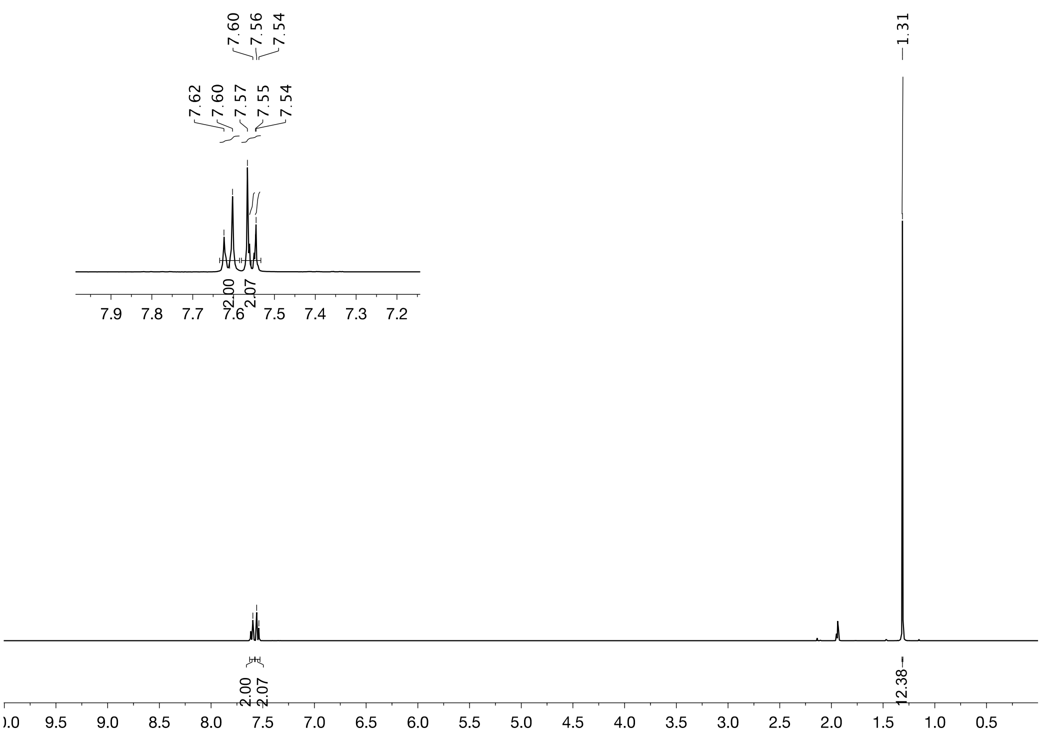

^{13}C NMR of 2-(4-Bromophenyl)-4,4,5,5-tetramethyl-1,3,2-dioxaborolane (3a)

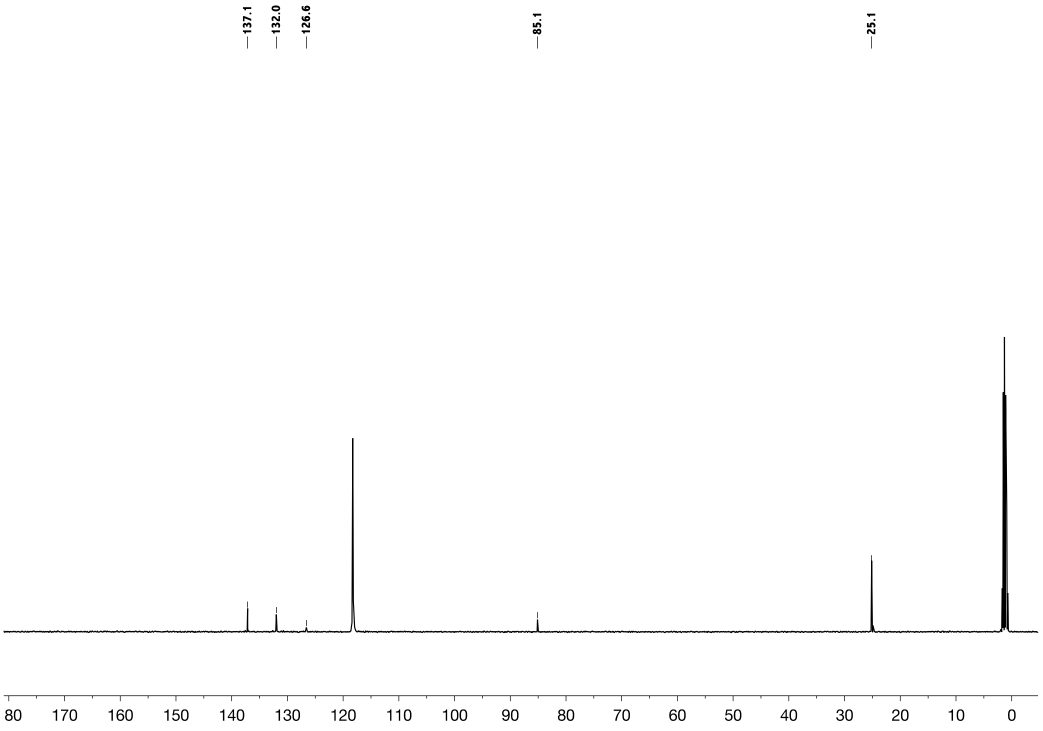

^{11}B NMR of 2-(4-Bromophenyl)-4,4,5,5-tetramethyl-1,3,2-dioxaborolane (3a)

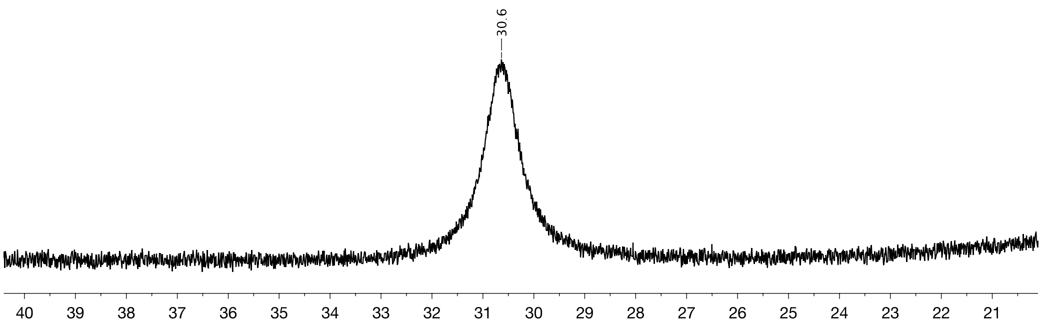

^{1}H NMR of 2-(4-Iodophenyl)-4,4,5,5-tetramethyl-1,3,2-dioxaborolane (3d)

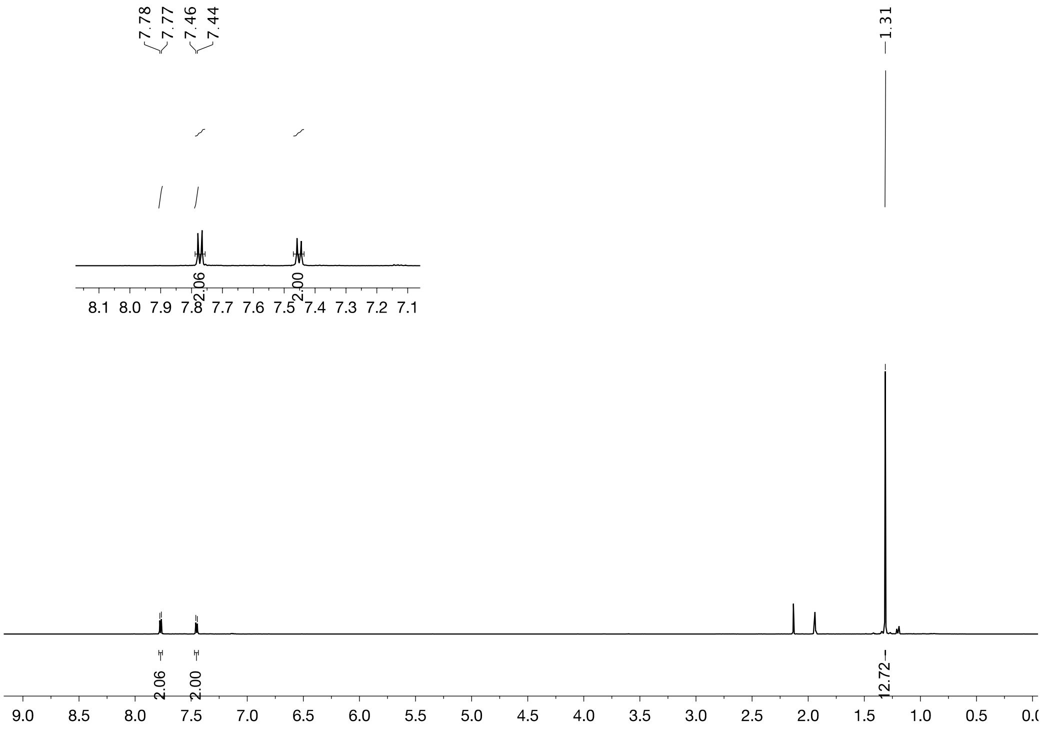

^{13}C NMR of 2-(4-Iodophenyl)-4,4,5,5-tetramethyl-1,3,2-dioxaborolane (3d)

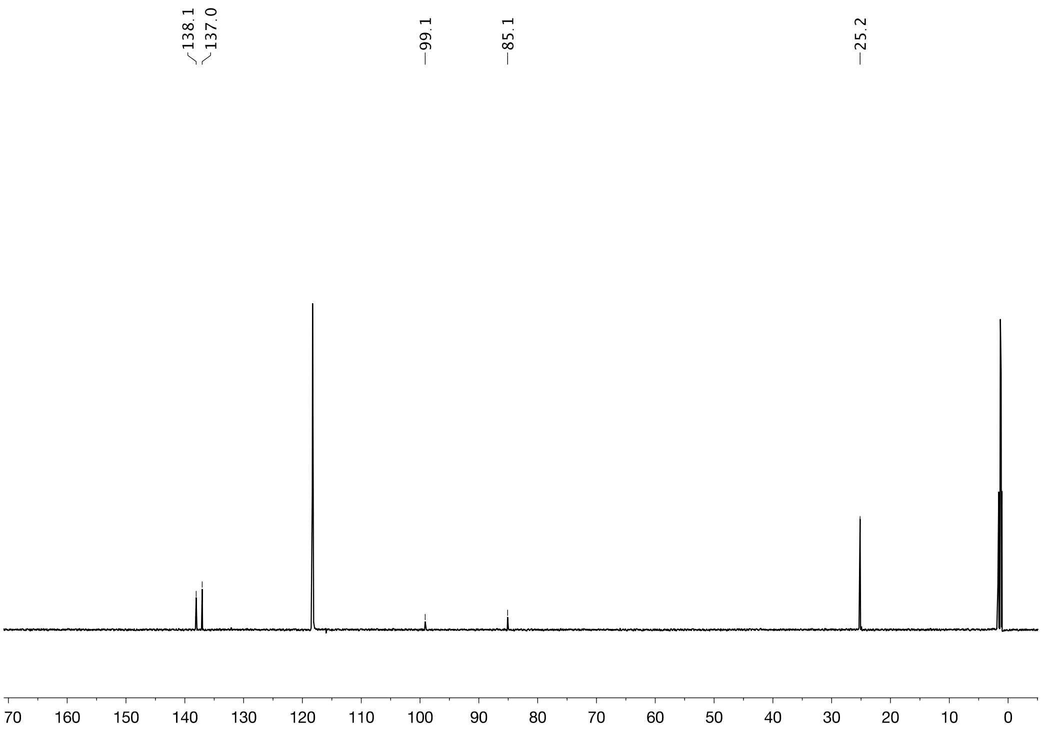

^{11}B NMR of 2-(4-Iodophenyl)-4,4,5,5-tetramethyl-1,3,2-dioxaborolane (3d)

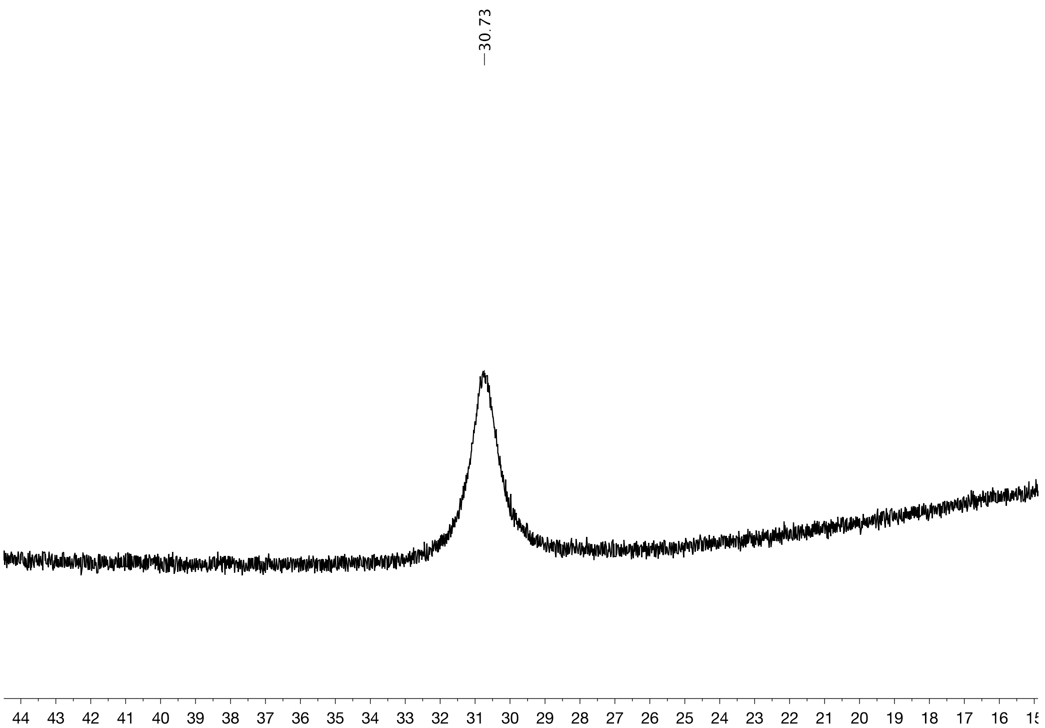

^{1}H NMR of 2-(4-Nitrophenyl)-4,4,5,5-tetramethyl-1,3,2-dioxaborolane (3e)

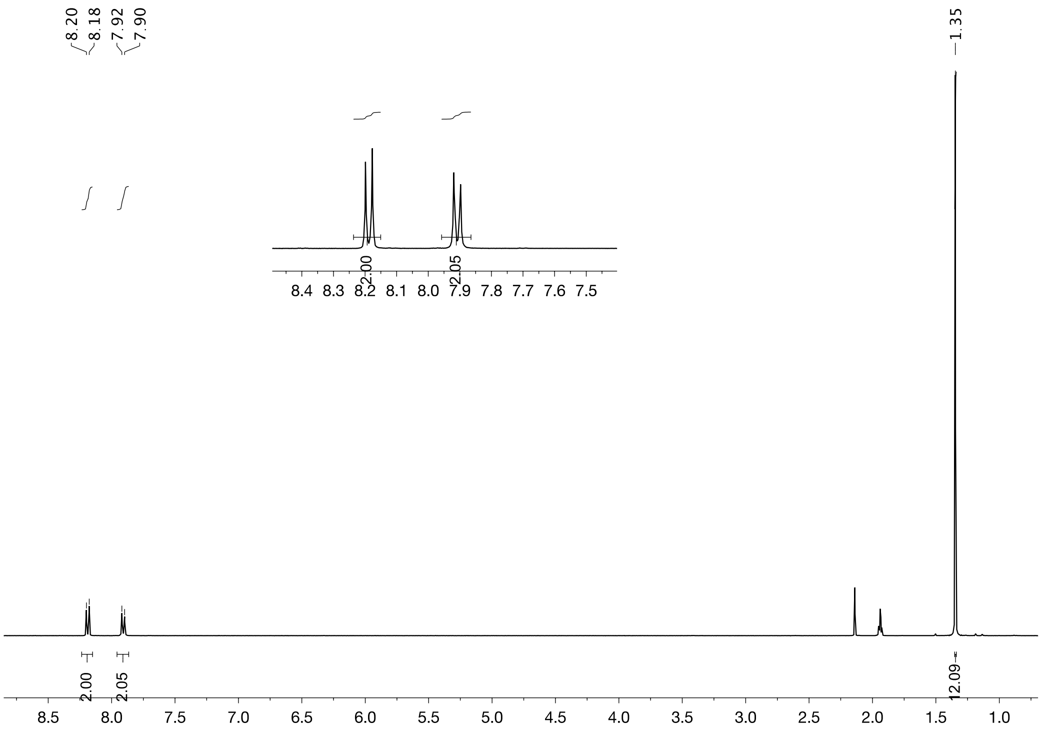

^{13}C NMR of 2-(4-Nitrophenyl)-4,4,5,5-tetramethyl-1,3,2-dioxaborolane (3e)

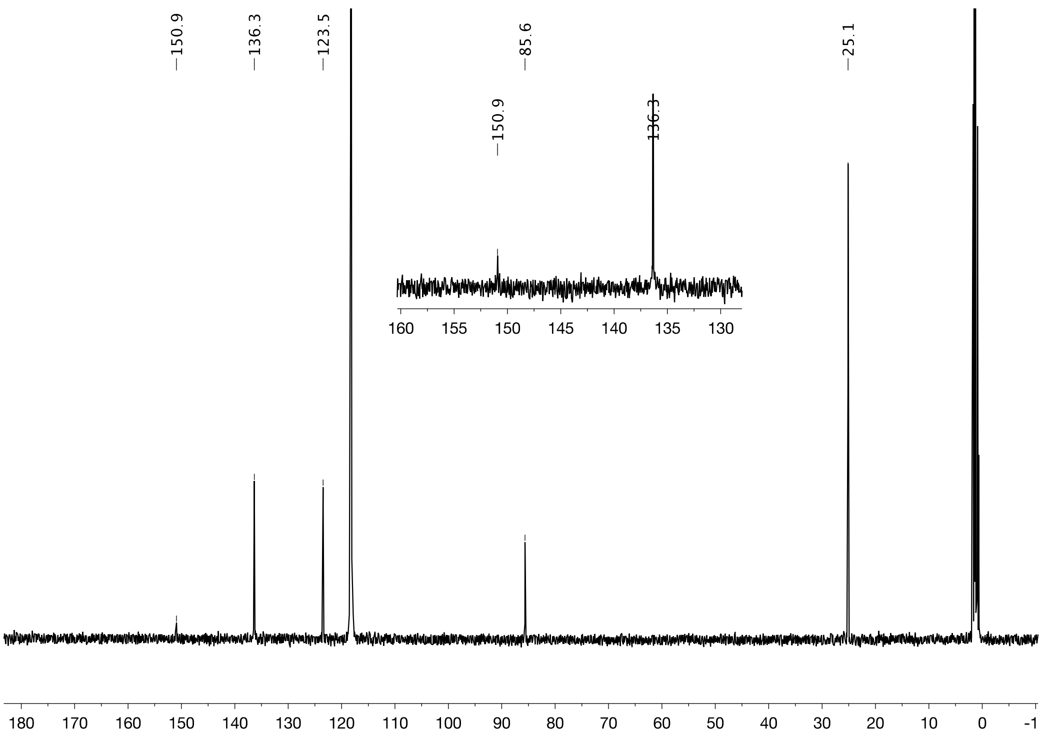

^{11}B NMR of 2-(4-Nitrophenyl)-4,4,5,5-tetramethyl-1,3,2-dioxaborolane (3e)

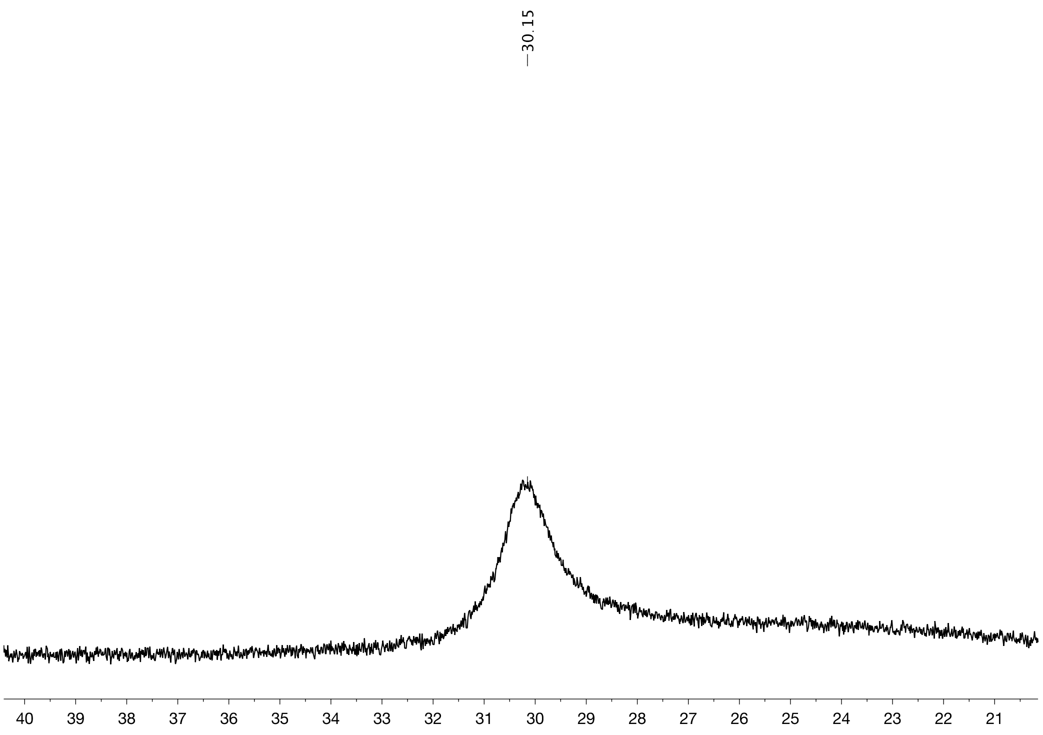

8. Differential scanning calorimetry (DSC) of 1a–e

4-Fluorophenyldiazonium tetrafluoroborate (1b)

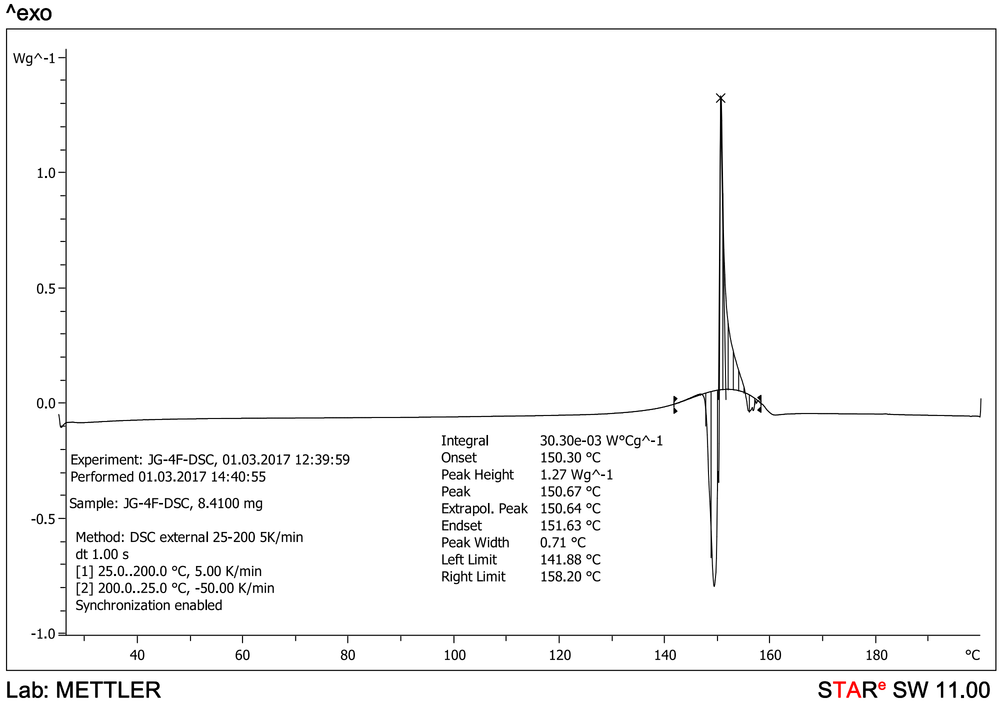

4-Chlorophenyldiazonium tetrafluoroborate (1c)

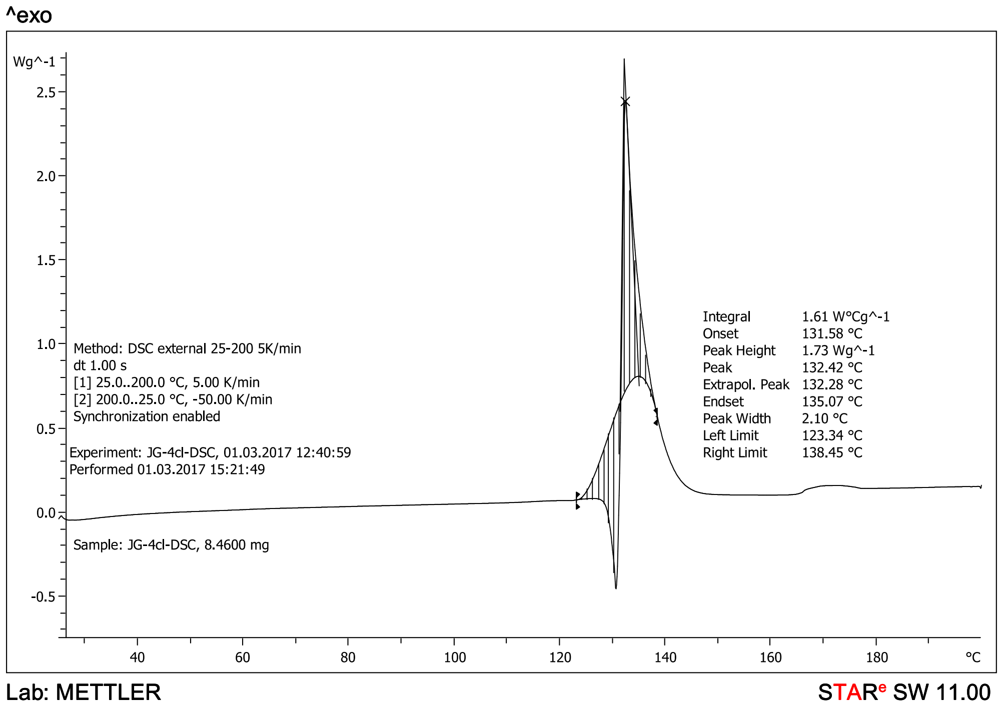

4-Bromophenyldiazonium tetrafluoroborate (1a)

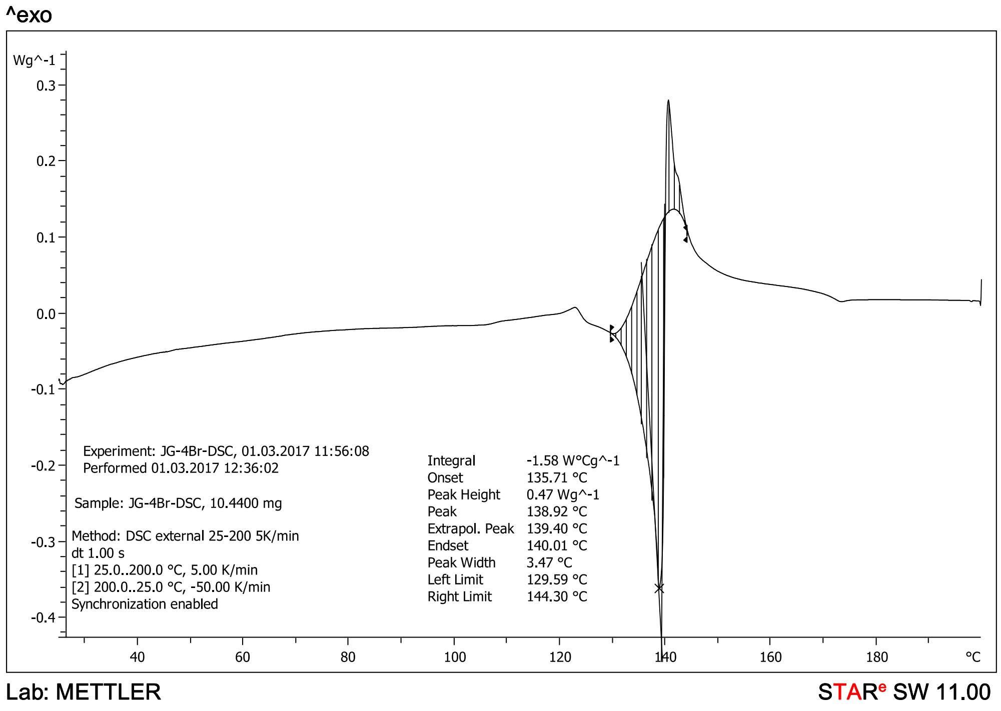

4-Iodophenyldiazonium tetrafluoroborate (1d)

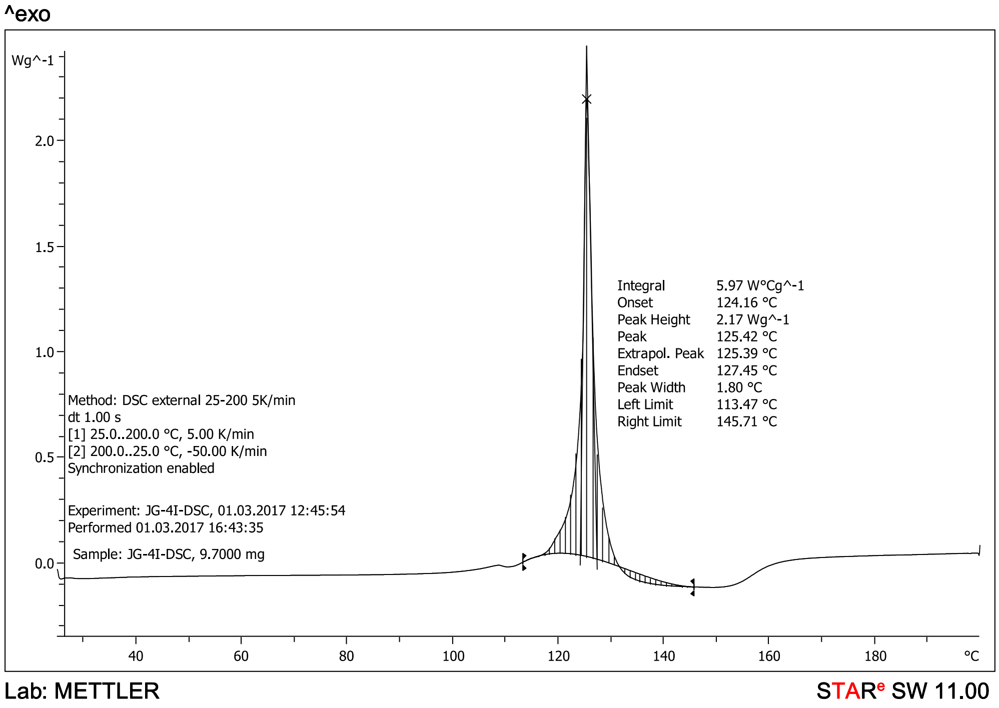

4-Nitrophenyldiazonium tetrafluoroborate (1e)

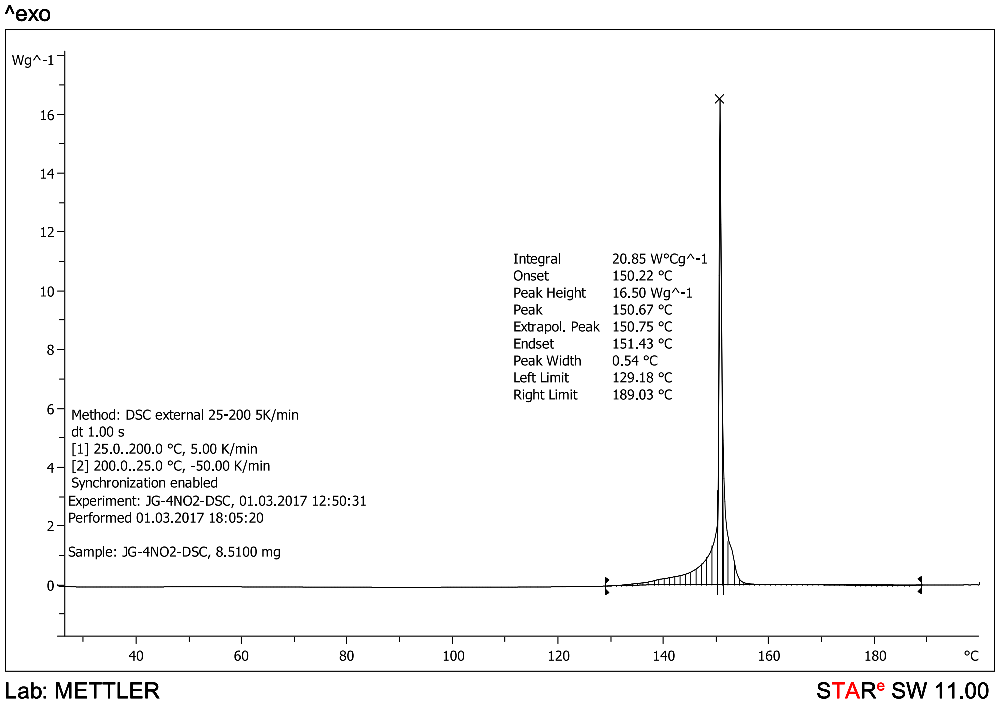

9. Thermal decomposition of iodophenyldiazonium tetrafluoroborate (1d)

100 mg of **1d** were weighted in a threaded screw cap glass test tube. The sample was heated in an oil bath for 10 min at 130 °C. Then the sample was cooled down and analyzed by ^{1}H NMR spectroscopy (see figure below). For a thermal decomposition of aryldiazonium salts observation by ageing, see ref. [5].

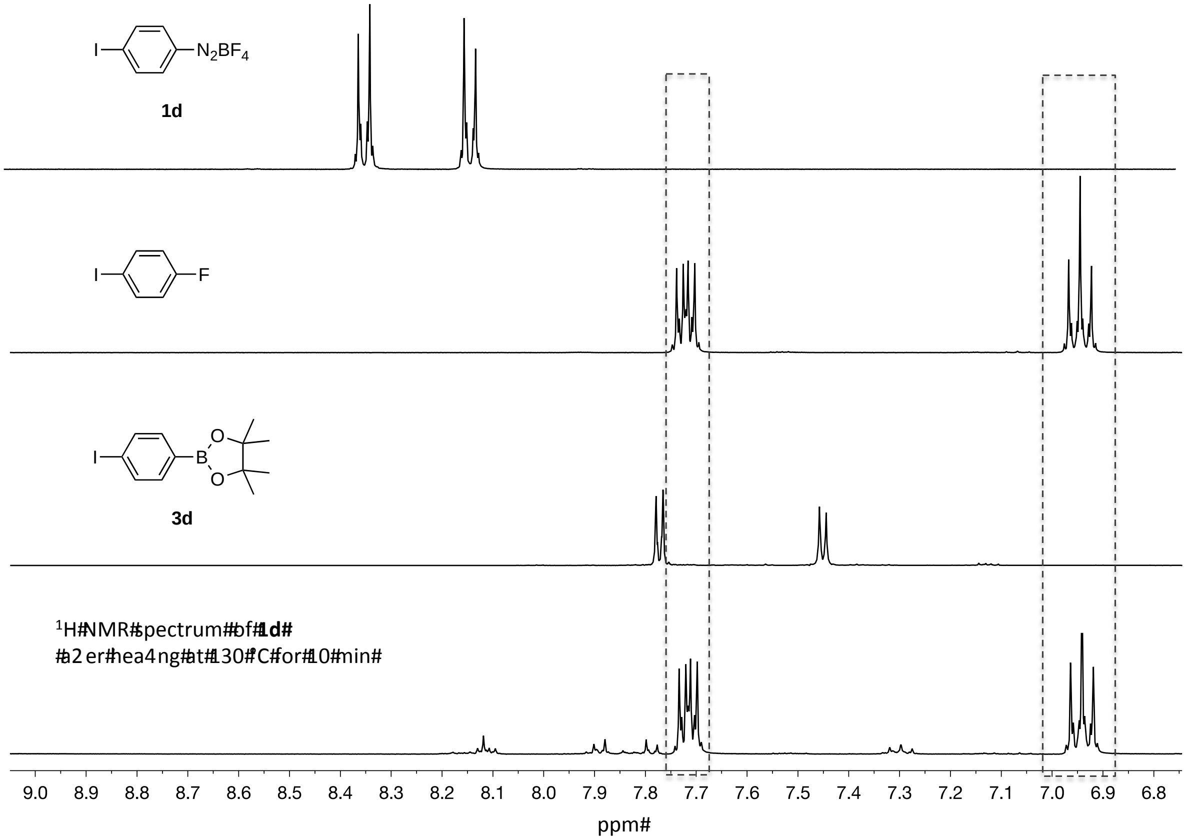

10. Borylation of 1a in the presence of 1,1-diphenylethene (4)

A mixture of **1a** (100 mg; 0.369 mmol), **2** (93.7 mg; 0.369 mmol) and **4** (66.4 mg; 0.369 mmol), eosin Y (11.96 mg; 5 mol%) was mixed for 1 h in a 25 mL PMMA milling jar with 15 ZrO_2 balls of 5 mm in diameter at 25 Hz. Irradiation of the reaction mixture was achieved by wrapping the milling jar with a blue-LED strip. After the milling was stopped, the reaction mixture was analyzed by gas chromatography-mass spectrometry.

Analysis by GC–MS of the reaction mixture of the borylation of 1a in the presence of 1,1-diphenylethene (4).

a) GC trace

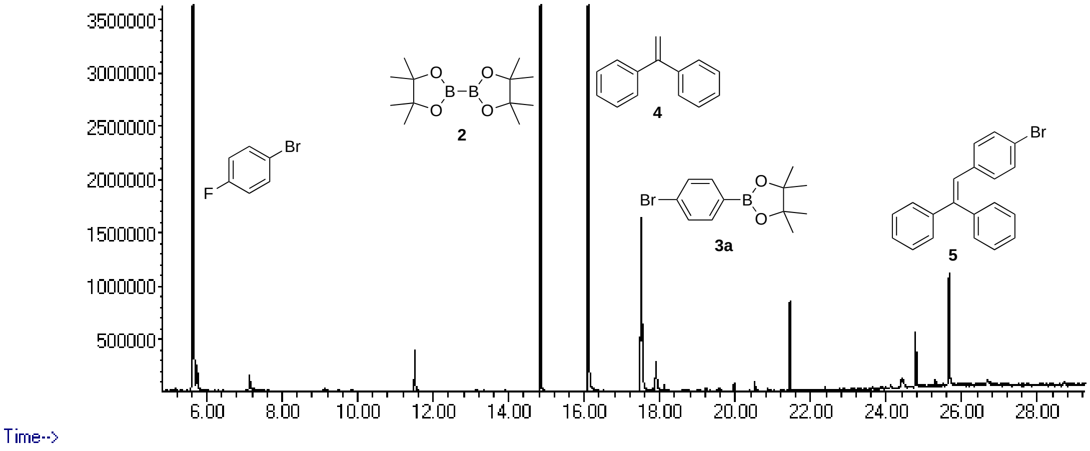

b) Mass spectrum of **3a** (retention time 17.54 min)

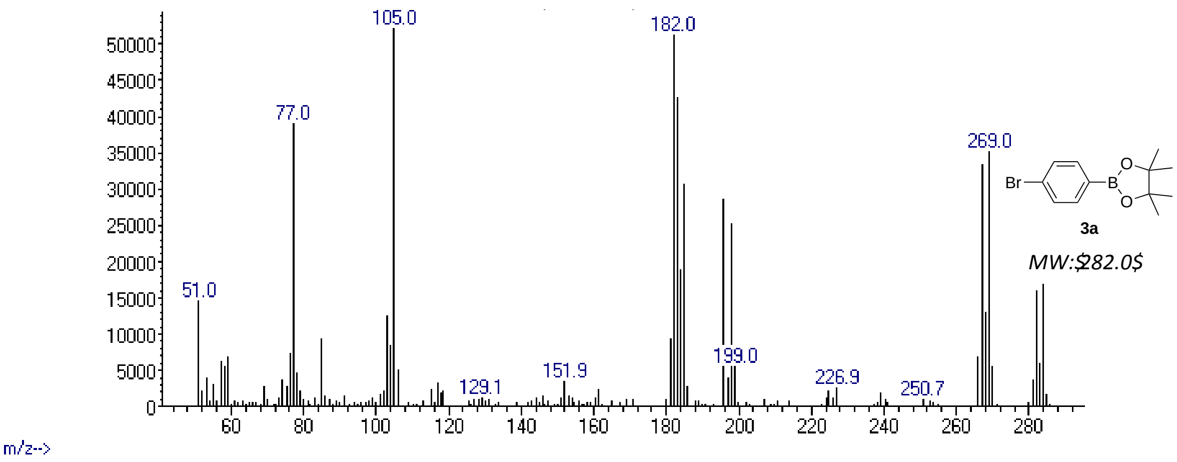

c) Mass spectrum of **5** (retention time 25.66 min)

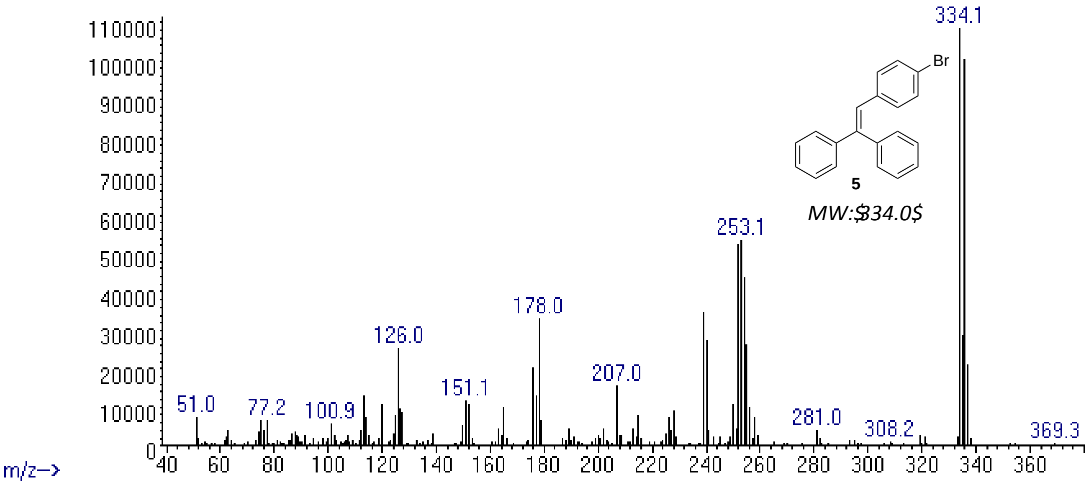

11. References

1. Bonin, H.; Delbrayelle, D.; Demonchaux, P.; Gras, E. *Chem. Commun.* **2010**, *46*, 2677.
2. Zhang, L.; Jiao, L. *J. Am. Chem. Soc*. **2017**, *139*, 607.
3. Xiao, T.; Dong, X.; Tang, Y.; Zhou, L. *Adv. Synth. Catal.* **2012**, *354*, 3195.
4. Bose, S. K.; Marder, T. B. *Org. Lett*. **2014**, *14*, 4562.
5. Majek, M.; Jacobi von Wangelin, A. *Acc. Chem. Res.* **2016**, *49*, 2316.
